# Intestinal Taxa Abundance and Diversity in Inflammatory Bowel Disease Patients: An Analysis including Covariates and Confounders

**DOI:** 10.3390/nu14020260

**Published:** 2022-01-08

**Authors:** Adelaide Teofani, Irene Marafini, Federica Laudisi, Daniele Pietrucci, Silvia Salvatori, Valeria Unida, Silvia Biocca, Giovanni Monteleone, Alessandro Desideri

**Affiliations:** 1 Department of Biology, University of Rome Tor Vergata, Via Montpellier 1, 00133 Rome, Italy; Adelaide.Teofani@uniroma2.it (A.T.); valeria.unida@gmail.com (V.U.); 2 Department of Systems Medicine, University of Rome Tor Vergata, Via Montpellier 1, 00133 Rome, Italy; irene.marafini@gmail.com (I.M.); federica.laudisi@gmail.com (F.L.); silviasalvatori23@gmail.com (S.S.); biocca@med.uniroma2.it (S.B.); 3 Department for Innovation in Biological, Agro-food and Forest Systems, University of Tuscia, 01100 Viterbo, Italy; daniele.pietrucci.89@gmail.com; 4 Institute of Biomembranes, Bioenergetics and Molecular Biotechnologies, IBIOM, CNR, 70126 Bari, Italy

**Keywords:** microbiota, ulcerative colitis, Crohn’s disease, diet, 16S rRNA

## Abstract

Intestinal dysbiosis has been widely documented in inflammatory bowel diseases (IBDs) and is thought to influence the onset and perpetuation of gut inflammation. However, it remains unclear whether such bacterial changes rely in part on the modification of an IBD-associated lifestyle (e.g., smoking and physical activity) and diet (e.g., rich in dairy products, cereals, meat and vegetables). In this study, we investigated the impact of these habits, which we defined as confounders and covariates, on the modulation of intestinal taxa abundance and diversity in IBD patients. 16S rRNA gene sequence analysis was performed using genomic DNA extracted from the faecal samples of 52 patients with Crohn’s disease (CD) and 58 with ulcerative colitis (UC), which are the two main types of IBD, as well as 42 healthy controls (HC). A reduced microbial diversity was documented in the IBD patients compared with the HC. Moreover, we identified specific confounders and covariates that influenced the association between some bacterial taxa and disease extent (in UC patients) or behaviour (in CD patients) compared with the HC. In particular, a PERMANOVA stepwise regression identified the variables “age”, “eat yogurt at least four days per week” and “eat dairy products at least 4 days per week” as covariates when comparing the HC and patients affected by ulcerative proctitis (E1), left-sided UC (distal UC) (E2) and extensive UC (pancolitis) (E3). Instead, the variables “age”, “gender”, “eat meat at least four days per week” and “eat bread at least 4 days per week” were considered as covariates when comparing the HC with the CD patients affected by non-stricturing, non-penetrating (B1), stricturing (B2) and penetrating (B3) diseases. Considering such variables, our analysis indicated that the UC extent differentially modulated the abundance of the *Bifidobacteriaceae*, *Rikenellaceae*, *Christensenellaceae*, *Marinifilaceae*, *Desulfovibrionaceae*, *Lactobacillaceae*, *Streptococcaceae* and *Peptostreptococcaceae* families, while the CD behaviour influenced the abundance of *Christensenellaceae*, *Marinifilaceae*, *Rikenellaceae*, *Ruminococcaceae*, *Barnesiellaceae* and *Coriobacteriaceae* families. In conclusion, our study indicated that some covariates and confounders related to an IBD-associated lifestyle and dietary habits influenced the intestinal taxa diversity and relative abundance in the CD and UC patients compared with the HC. Indeed, such variables should be identified and excluded from the analysis to characterize the bacterial families whose abundance is directly modulated by IBD status, as well as disease extent or behaviour.

## 1. Introduction

Inflammatory bowel diseases (IBDs), which include Crohn’s disease (CD) and ulcerative colitis (UC), are chronic immune-mediated disorders of the gastrointestinal tract of unknown aetiology. A large body of evidence suggests that, in IBD, the pathological process results from the interaction between genetic and environmental factors, which promotes an abnormal immune response against a component of normal intestinal flora [1,2,3]. Intestinal dysbiosis, which is characterized by the loss of beneficial bacteria, overgrowth of potentially pathogenic bacteria and loss of bacterial diversity, has been widely documented in IBD and is thought to influence the onset and perpetuation of gut inflammation [4,5,6,7,8]. Several factors may contribute to intestinal dysbiosis, such as host genetics, concomitant medications and lifestyle. Dietary habits are known to deeply shape gut microbiota composition [9,10,11,12]. The fact that IBD has a high prevalence in countries with “Westernized” dietary habits supports a direct relationship between flora composition and diet [13,14,15]. High-fat, high-sugar and low-fibre diets reduce the richness of gut microbiota and promote the expansion of pathogenic species [16,17,18]. Food additives, which are highly prevalent in the Western diet, also impair gut homeostasis and promote intestinal inflammation [19,20,21,22,23].

In this context, it is also noteworthy that modification of the patient’s usual diet by excluding food groups, restricting or adding dietary components, is often used to mitigate the ongoing inflammation and/or attenuate IBD-related symptoms [24]. These observations raise the possibility that some of the bacterial changes seen in IBD may rely on specific lifestyle and dietary habits.

The aim of this study was to analyze the potential role of covariates and confounders in modulating the taxa diversity and relative abundances in IBD.

## 2. Materials and Methods

### 2.1. Patients and Samples

One hundred and ten patients with a confirmed diagnosis of IBD and 42 HC were prospectively recruited between April 2019 and February 2020 at a single university hospital (University of Rome Tor Vergata). Participants, aged between 19 and 72 years old, provided faecal samples collected in Pre-Analytical Sample Processing (PSP) stool collection tubes (Invitek Molecular). For each patient, the following variables were collected: gender, age, smoking habit, anthropometric parameters, disease extent and behaviour. Disease characteristics were defined according to the Montreal classification. Patients with a history of infectious disease in the last three months and who had been using antibiotics, nonsteroidal anti-inflammatory drugs (NSAIDs) or proton pump inhibitors in the last 4 weeks before enrolment were excluded. Disease activity was assessed using the Harvey–Bradshaw index for CD patients (remission: *n* = 39, mild activity: *n* = 8, moderate activity: *n* = 5) and partial Mayo Clinic scores for UC patients (remission: *n* = 35, mild activity: *n* = 19, moderate activity: *n* = 4). Faecal calprotectin (fCal) levels were analyzed from 28 CD patients (fCal > 250 mg/kg in 6 patients) and 34 UCpatients (fCal > 250 mg/kg in 14 patients). Information about the patients’ lifestyle and dietary habits was collected through a questionnaire modified by an anamnestic interview applied in a previous study [25], where the effects of confounders and covariates modulating the differential abundance of microbial species between Parkinson’s disease patients and healthy controls was analyzed. The anamnestic interview, modified according to the Italian diet and lifestyle habits, was successfully used in our previous work to assess the role of possible confounders and covariates in a selected population of Italian patients affected by Parkinson’s disease [26] and it was applied in the present study. All patients gave informed consent and the study was approved by the local ethics committee.

### 2.2. Sequencing and Bioinformatic Analysis of 16S rRNA Amplicons

Faecal samples were collected using PSP stool collection tubes (Invitek Molecular) containing 8 mL of Stool DNA Stabilizer. DNA extraction from the stool samples was performed with PSP Spin Stool DNA Kit Plus (Invitek Molecular) following the manufacturer’s instructions. The purified DNA was quantified using a NanoDrop spectrophotometer ND1000 (Termofisher). 16S rRNA amplicon (V3–V4 regions) sequencing analysis was performed using an Illumina MiSeq 2×300bp. The quality of the raw sequences was checked using FastQC software and the primers, together with the adapters, were removed using Cutadapt, while the QIIME 2 pipeline was used to analyse the preprocessed reads [27]. In detail, reads were chimaera-checked and clustered in amplicon sequence variants (ASVs) using the DADA2 algorithm [28]. The q2-feature-classifier and the Silva database vr. 138 were used for taxonomic assignment of representative sequences obtained by DADA2 [29,30]. Statistical analyses of the ASV tables were performed in R using phyloseq, DESeq2, vegan and ggplot packages; ASVs with low frequencies were removed from the analyses [29] and data that were normalized by DESeq2 [31,32,33] were used to measure the α- and β-diversity metrics. The tax_glom function in the phyloseq package was used to sum up the data to different taxonomic levels and compare between samples.

### 2.3. Statistical Analysis

We performed the statistical analyses in the R 3.6.1 environment, using the vegan 2.5.6 and phyloseq 1.30.0 packages. Sample data were normalized by the DESeq2 R package, which is widely used to normalize data from 16S sequencing [33] and adopts a normalization method in order to estimate the dispersion across genes when the sample size is small [31]. We filtered OTUs by their prevalence, considering only those present in at least 10% of the samples. Alpha-diversity was computed at the species level using three metrics: Chao1, Shannon and Simpson. Beta-diversity was computed using four metrics: Bray–Curtis and weighted, unweighted and generalized Unifrac metrics. In our analysis, we considered both covariate and confounder variables as possible biasing factors and we assessed for their marginal effects using a generalized linear model (GLM), as previously applied [26]. Covariates are those variables that explain part of the variability in the outcome, while confounders may distort or mask the effects of the target variable (IBD status) on the outcome. We looked for covariates and confounders between 16 lifestyle and diet variables collected from participants. To find unbalanced variables, we used the Kruskal–Wallis test (non-parametric method) for numerical variables, while the Fisher’s exact test for the categorical variables and variables with a *p* < 0.05 were considered as potential confounders. The covariates were identified using the PERMANOVA test with 9999 permutations, assessing each variable’s marginal effect. For each of the four metrics used in measuring the β-diversity, we identified microbiota-shaping variables using a stepwise regression through manual backward elimination. We considered all the variables that were statistically significant (*p* < 0.05) for at least one metric, besides the target variable, as covariates. The PERMANOVA test was also used to confirm the effect of IBD status on the microbiome composition. The GLM multivariate analysis, adjusting for confounders and covariates, allows the comparison between unbalanced groups and to consider the part of variability explained by the covariates. We evaluated the differential abundance of each taxon by fitting four distributions: negative binomial, zero-inflated negative binomial, Poisson and hurdle distribution (using R packages: stats 3.4.4, MASS 7.3–50 and pscl 1.5.2.), and we chose the model with the lowest Bayesian information criteria score.

In a first approach, which we named the IBDCC model, for each distribution, we compared a “full” model with a “nested” model. The “full” GLM described the taxon behaviour, taking into account the target variable and all the covariates and confounders previously identified:

Taxon ~ IBD status + sex + age + yogurt + dairy products + cereals + fruits and vegetables + legumes + bread.

The “nested” model instead described the taxon behaviour by only considering the covariates and confounders:

Taxon ~ sex + age + yogurt + dairy products + cereals + fruits and vegetables + legumes + bread.

In a second approach, called the IBD model, where the role of the covariates and confounders were not taken into account, for each distribution, we compared a “full” model, which only included the IBD status, with a “null” model, which included a constant (i.e., 1). We compared the “full” model with the “nested” or the “null” model using the ANOVA function in R and corrected the *p*-values for multiple testing using the Benjamini–Hochberg procedure. We considered only those with a *p* < 0.05 as differentially abundant taxa.

## 3. Results

### 3.1. Microbiota Diversity Analysis, Study Cohort Characteristics and Identification of Covariates and Confounders

We collected 152 faecal samples from 52 CD patients, 58 UC patients and 42 HC. Genomic DNA was extracted from all samples and the 16S rRNA V3-V4 regions were amplified. The α-diversity was statistically significant for all the three indices (*p* = $4.401\times 10^{-9}$ for Chao1, *p* =$\;3.650\times 10^{-9}$ for Shannon, *p* = $1.617\times 10^{-7}$ for Simpson), indicating a reduced microbial diversity for the CD and UC patients compared with the HC, as previously reported [34,35]. Results from the microbiome structure and composition in IBD patients and HC were assessed by using a PERMANOVA test with four β-diversity metrics (Bray–Curtis and weighted, unweighted Unifrac and Canberra metrics).

Since environmental factors could act as confounders, affecting the differential abundance analysis of bacterial species and causing spurious associations, we provided each participant with a specific questionnaire to gather information about their lifestyle and dietary habits. We observed that some variables were skewed among the three groups. In particular, four variables, namely “eat dairy products at least 4 days per week”, “eat fruit and vegetables at least 4 days per week”, “eat cereals at least 4 days per week” and “eat legumes at least 4 days per week”, were found to be unbalanced in the UC and CD patients compared with the HC and were therefore considered as possible confounders (Table A1). A PERMANOVA test considering only IBD status as a grouping variable indicated that this condition was a statistically significant predictor of gut microbiota composition for all the metrics (*p*-value < 0.0001 for Bray–Curtis, Canberra and weighted and unweighted Unifrac). Results from another PERMANOVA test showed that the microbiota differences between the CD and HC were still significant when considering all the variables reported in Table A1. Moreover, these data also indicated that five variables, namely “age”, “gender”, “eat bread at least 4 days per week”, “eat yogurt at least 4 days per week” and “eat dairy products at least 4 days per week”, were statistically significant for at least one metrics. Therefore, these variables were considered as covariates since they explained part of the variability in the microbiota structure (Table 1). Given that both confounders and covariates may introduce biases in the differential abundance analysis, we assessed their relative importance in the analysis of the faecal microbiota composition in the UC and CD patients and the HC. 

### 3.2. Differential Abundance Analysis between IBD and HC Taxa

**Figure 1:**
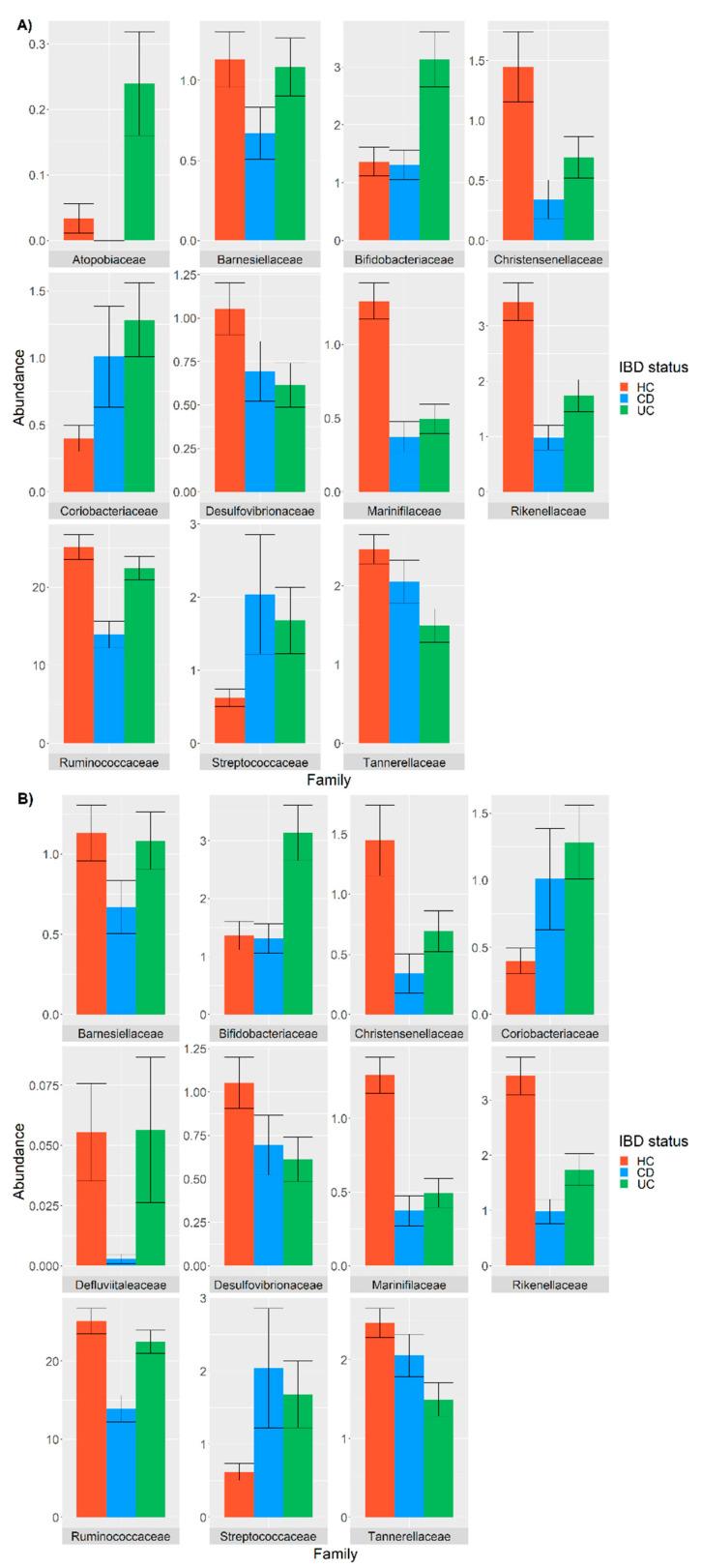
Differentially abundant bacterial families in samples from the CD (cyan) and UC patients (green) compared with the HC (pink), as detected using a GLM model only considering the IBD status (**A**) or using a GLM considering the IBD status and covariates and confounders (**B**). The relative abundance is plotted in log10 on the *y*-axis.

**Figure 2:**
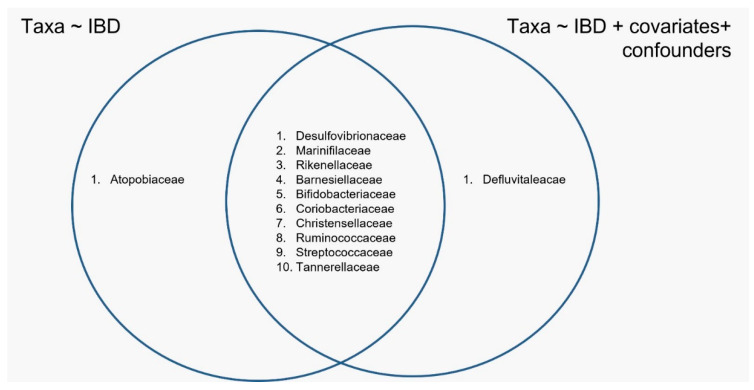
Venn diagram indicating the bacterial families identified using the IBD and IBDCC models.

A generalized linear model (GLM) was used to evaluate the influence of IBD status on taxa abundance, comparing a GLM where the IBD status was the only possible variable (IBD model) with a GLM that was a linear combination of IBD status, covariates and confounders (IBDCC model). The analysis indicated that the bacterial families that were differentially abundant in the CD and UC patients compared with the HC were different when considering the IBD and IBDCC models (Figure 1 and Figure 2). The two methods converged in identifying a different abundance of 10 bacterial families (Figure 2), but the difference in the abundance of the *Atopobiaceae* family was found to be significant only when testing the IBD model, while the IBDCC model indicated that *Defluvitaleacae* was the only family to be differentially abundant in IBD samples compared with the HC (Figure 2).

Concerning the commonly identified families, *Coriobacteriaceae* and *Streptococcaceae* showed a higher abundance in both the CD and UC patients compared with the HC, while reduced frequencies of *Christensenellaceae*, *Desulfovibrionellaceae*, *Marinifilaceae*, *Rikenellaceae*, *Ruminococcaceae*, *Tannerelleaceae* and *Barneselliaceae* were observed in the CD and UC patients compared with HC (Figure 1). Moreover, the frequency of *Atopobiaceae*, *Bifidobacteriaceae* and *Defluvitillaceae* were increased in the UC patients and decreased in the CD patients compared with the HC (Figure 1).

The *Atopobiaceae* and *Defluvitaleacae* families, named as “non-overlapping families” (NOFs), were investigated to identify the influence of one or more variables on their relative abundance.

### 3.3. Analysis of the Covariates and Confounders Influencing Taxa Abundance

We tested the influence of each covariate and confounder related to lifestyle and dietary habits on the abundance of the two NOFs using two different approaches. In the first one, we compared the IBD model with a linear model considering the IBD status plus an additional single variable. In the second approach, we compared the IBDCC model with a linear model that included the IBD status plus all variables except one, which was excluded in an iterative procedure. The first approach showed that the differential abundance of the *Atopobiaceae* family among the UC, CD and HC groups was not significant when the condition “eat cereals at least 4 days per week” was included in the model. In line with these results, the difference between the UC, CD and HC groups observed for the *Atopobiaceae* family was not any more significant when excluding “eat cereals at least 4 days per week” from the covariates by using the second approach (Table 2). These results indicated that “eat cereals at least 4 days per week” strongly impacted the *Atopobiaceae* abundance. In line with our data, it was recently reported that cereals can perturb *Atopobiaceae* family abundances [36].

The same approach, which was applied to analyse the abundance of the *Defluvitaleacae* family, indicated that this family was not differentially modulated by a single variable, but by a combination of several variables.

### 3.4. Association between Gut Microbiota and Montreal Classification

We next assessed whether the frequency of specific bacterial families correlated with the extent and behaviour of the disease using the IBDCC model. The UC extent was classified using the Montreal classification as ulcerative proctitis (E1), left-sided UC (distal UC) (E2) and extensive UC (pancolitis) (E3). In contrast, the CD behaviour was classified using the Montreal and Vienna classification as non-stricturing, non-penetrating (B1), stricturing (B2) and penetrating (B3) [37]. The analysis, which was carried out on 42 HC, 9 E1, 18 E2 and 28 E3 samples for the UC patients and on 42 HC, 27 B1, 22 B2 and 3 B3 samples for the CD patients, indicated that alpha-diversity was statistically significant for both classifications for all the three indices: *p* = $\;9.881\times 10^{-5}$ (Chao1), *p* = $3.499\times 10^{-5}$ (Shannon) and *p* = $5.763\times 10^{-4}$ (Simpson) for the E1, E2 and E3 groups, respectively, compared with the HC; and *p* = $7.166\times 10^{-8}$ (Chao1), *p* = $7.445\times 10^{-8}$ (Shannon) and *p* =$\;1.220\times 10^{-6}$ (Simpson) for the B1, B2 and B3 groups, respectively, compared with the HC. A PERMANOVA test confirmed that the gut microbiota composition was a good predictor for the UC extent and CD behaviour.

Table A2 shows that three variables, namely, “lost 5 kg in the last year”, “eat dairy products at least 4 days per week” and “drink alcohol at least 4 days per week”, were significantly unbalanced across the E1, E2, E3 and HC groups, while Table A3 indicates that four variables, namely, “lost 5 kg in the last year”, “gained 5 kg in the last year”, “eat bread at least 4 days per week” and “eat legumes at least 4 days per week”, were significantly unbalanced across the B1, B2, B3 and HC groups. A PERMANOVA stepwise regression identified the variables “age”, “eat yogurt at least 4 days per week” and “eat dairy products at least 4 days per week” as covariates for the E1, E2 and E3 groups, while the variables “age”, “gender”, “eat meat at least 4 days per week” and “eat bread at least 4 days per week” were considered as covariates for the B1, B2 and B3 groups.

**Figure 3:**
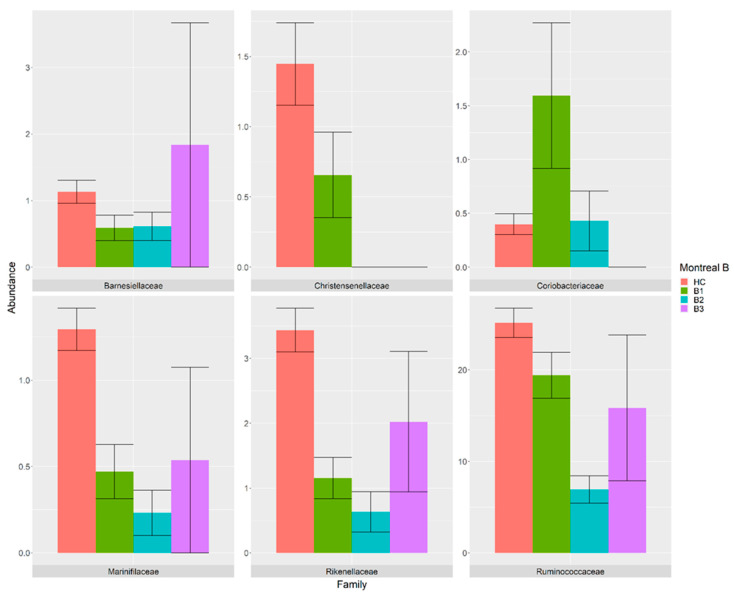
Bacterial families that were differentially present in the samples from the B1 (green), B2 (cyan) and B3 (purple) patients compared with the HC (pink). The relative abundance is plotted in log10 on the *y*-axis.

**Figure 4:**
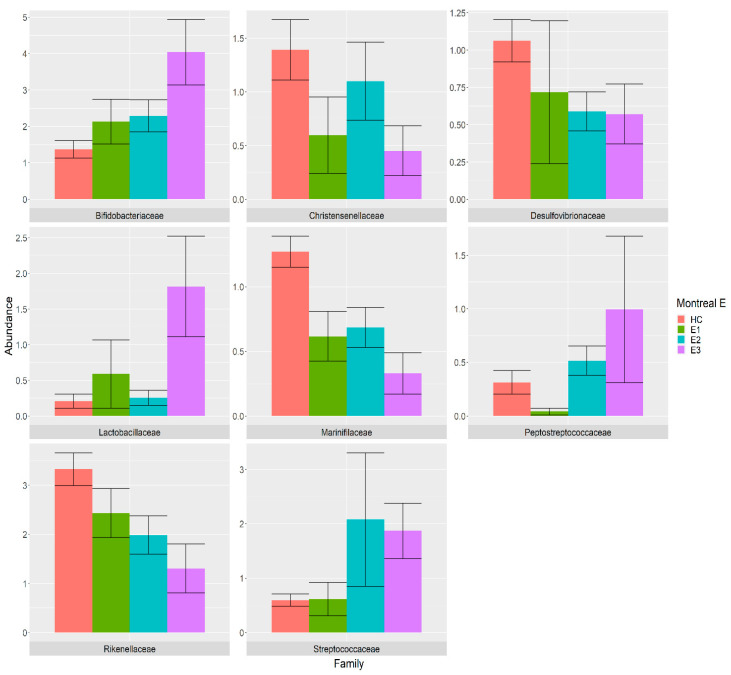
Bacterial families that were differentially abundant in samples from the E1 (green), E2 (cyan) and E3 (purple) patients compared with the HC (pink). The relative abundance is plotted in log10 on the *y*-axis.

The influence of the UC extent and CD behaviour on the taxa abundances, considering their covariates and confounders, indicated that the UC extent differentially modulated the abundances of eight bacterial families (Figure 3), while the CD behaviour influenced the abundance of six bacterial families (Figure 4) compared with the HC. Among them, the *Christensenellaceae* family was consistently decreased in the B1 class when compared with HC and absent in the B2 and B3 classes. Similarly, the *Marinifilaceae*, *Rikenellaceae* and *Ruminococcaceae* families were decreased in the B1, B2 and B3 classes compared with the HC. In contrast, the relative abundance of *Barnesiellaceae* was found to be decreased in the B1 and B2 classes compared with the HC, while it was increased in the B3 class. Interestingly, the abundance of the *Coriobacteriaceae* family was found to be higher in the B1 class, similar in the B2 class and absent in the B3 class compared with the HC (Figure 3).

Among those families that were differentially abundant among the UC extent classes, the *Bifidobacteriaceae* family showed a clear increasing trend in the HC, E1, E2 and E3 groups, while *Desulfovibrionaceae* and *Rikenellaceae* showed decreasing trends compared with the HC. The abundance of the *Christensenellaceae* and *Marinifilaceae* families were decreased in the E1, E2 and E3 classes compared with the HC, while the *Lactobacillaceae* abundance was greatly increased in the E3 class compared with HC. Moreover, the frequency of *Streptococcaceae* family was increased in the E2 and E3 classes compared with the HC, while *Peptostreptococcaceae* was found to be decreased in the E1 class and increased in the E2 and E3 classes compared with the HC (Figure 4).

## 4. Discussion

In this study, we evaluated the differences in microbiota structure and composition in faecal samples isolated from IBD patients (52 CD, 58 UC) and healthy controls (42 HC) using 16S rRNA sequencing, considering the contribution of covariates and confounders that may bias the associations between microbiota composition and the pathological status. We focused our attention on the effect of common confounding factors (age, gender, diet) since they are known to deeply impact gut microbiota composition [38,39,40,41,42,43]. We confirmed that the variables “age”, “gender” and “dietary habits” influenced the variance of the gut microbiota and the taxa abundance across cases (Table A1). In particular, our data indicated that bread consumption could modulate the abundance of some microbial species, in line with previous studies showing that the consumption of industrial bread induced the proliferation of Bacteroides, leading to systemic inflammation [44]. In order to identify potential variables that are able to influence the microbiota composition independently of the disease, we compared two generalized linear models: the IBD model, which did not consider the contribution of confounders and covariates, and the IBDCC model, which included confounders and covariates. Interestingly, our results showed the differential abundance of the *Atopobiaceae* family, identified by the IBD model, was no more significant when using the IBDCC model, indicating that its abundance was strictly dependent on the variable “eat cereals at least 4 days per week”. In line with this observation, a significant increase in the *Atopobium* genus was previously reported to correlate with the consumption of wholegrain products [36]. In contrast, the differential abundance of the *Defluvitaleacae* family was not modulated by a single variable, suggesting that a combination of more than a single external factor was necessary to influence its frequency in the IBD patients compared with the HC.

However, both the IBD and IBDCC models converged to identify a differential abundance of 10 bacterial families, thus confirming a crucial role of the disease in modulating the microbiota composition. In particular, the models identified a lower abundance of the *Christensenellaceae*, *Ruminococcaceae*, *Rikenellaceae* and *Tannerellaceae* families in IBD patients, which are butyrate-producing bacteria that are able to protect the host from gut inflammation and IBD exacerbation, as previously described [45,46,47,48,49]. We also observed a higher abundance of the *Coriobacteriaceae* family in both the CD and UC samples compared with the HC, thus confirming previous studies [49]. A possible explanation could be that some members of this family (e.g., *A. parvolum*) can produce hydrogen sulfide, thus promoting rapid and severe pancolitis [50]. We reported a reduction in the *Desulfovibrionaceae* family in our UC and CD samples compared with the HC, in contrast with some previous studies [51]. This discrepancy may have been due to the administration of 5-aminosalicylic acid-based drugs in some patients, which may have affected the activity of sulfate-reducing bacteria [52]. We also observed a higher frequency of the *Bifidobacteriaceae* family in the UC samples compared with the CD and HC samples, in contrast with previous observations [49,53]. The abundance of *Bifidobacterium* is known to be strictly dependent on the disease phase [54], thus providing a possible explanation for the discrepancy between our results and previous observations. Moreover, we found that the *Streptococcaceae* family was increased in both the UC and CD samples compared with the HC, in line with other studies [49,55].

The association between frequencies of microbial species and the Montreal classification of IBD was investigated by comparing 42 HC samples with 58 UC samples organized into E1 (*n* = 9), E2 (*n* = 18) and E3 (*n* = 28) classes and comparing 42 HC samples with 52 CD samples organized into B1 (*n* = 27), B2 (*n* = 22) and B3 (*n* = 3) classes. The alpha-diversity was decreased in the UC and CD samples compared with the HC group depending on the disease extent and behaviour, respectively. Using a GLM model corrected for covariates and confounders, we found six differentially abundant families in the CD classes and eight families in the UC classes when compared with the HC (Figure 3 and Figure 4).

## 5. Conclusions and Limitations of the Study

Multivariate models are the most reliable statistical methods used to identify and adjust for several factors related to a patient’s lifestyle and habits, allowing for handling several covariates and confounders simultaneously. Indeed, the most promising strategy used to properly correlate a pathological status to microbiota composition should match the case and controls participants with confounding variables, reducing the underlying noise derived from factors independently from the pathological status [56]. In this work, we showed that a multivariate GLM model allowed for identifying the microbial community variation according to the disease status. However, we must be aware that the characterization of human microbiota composition, achieved by comparing two different experimental conditions, is still challenging. The analysis of the microbiota composition of healthy patients and patients affected by a disease leads to more reliable results when including the effect of different habits and diets, even if there is still a part of the variance that remains unexplained. We underline the importance of choosing appropriate confounders and covariates to better suit the characteristics of the population in the study, i.e., Mediterranean diet variables in the case of the Italian population. However, the use of a highly specific habits questionnaire introduced difficulties in transferring the research results to different populations. 

Another important comment concerns the results obtained in this study comparing the different Montreal classification classes. We want to point out that the results showing alteration in specific families between the different Montreal scales were obtained by comparing groups made up of a small number of patients. The data were analyzed using the DESeq2 package, which adopts a normalization method to estimate the dispersion across genes when the sample size is small. However, the study still suffered from a limited number of samples and the final results must be confirmed by considering a larger sample size.

## Figures and Tables

**Table 1:** Regression PERMANOVA using four metrics to evaluate the β-diversity and identify microbiota shaping variables using a stepwise regression through manual backward elimination. The symbols indicate the *p*-value threshold (‘’*”: *p*-value ≤ 0.05, “**”: *p*-value ≤ 0.01 and “***”: *p*-value ≤ 0.001).

	Bray–Curtis	Unweighted Unifrac	Weighted Unifrac	Canberra
IBD	0.0001 ***	0.0001 ***	0.0001 ***	0.0001 ***
Age	0.0473 *			0.0265 *
Gender	0.0399 *		0.0052 **	
Bread		0.0304 *		
Yogurt				0.0367 *
Dairy products				0.0178 *

**Table 2:** Analysis of the variables’ (covariates and confounders) impact on bacterial families that were differently identified using the IBD and IBDCC models. The significance of the *Atopobiaceae* family was related to the presence of the “cereals” variable. The symbol “*” indicates a *p*-value ≤ 0.05.

Model	*Atopobiaceae*
IBD + cereals	*N*.*S*.
IBD + covariates + confounders (not including cereals)	0.0236 *
IBD + covariates + confounders (including cereals)	*N*.*S*.
IBD	0.0147 *

## Data Availability

The datasets used and/or analyzed during the current study are available from the corresponding author upon reasonable request.

## Appendix A

**Table A1:** Demographic and habit-related parameters of the cohort. The *p*-values refer to the Kruskall–Wallis test and Fisher’s test between IBD and the numerical or categorical variables, respectively. *n* = no, Y = yes. The variables in bold were significantly unbalanced between the HC (42 samples), CD (52 samples) and UC (58 samples) groups.

	*p*-Value	HC	CD	UC
Age	0.26	Mean: 38.02	Mean: 42.42	Mean: 43.43
		Median: 32.5	Median: 42	Median: 42
		Std.Dev: 15.95	Std.Dev: 15.95	Std.Dev: 14.04
		Min: 22	Min: 19	Min: 19
		Max: 72	Max: 67	Max: 69
Gender	0.21	F = 0.64,	F = 0.47,	F = 0.5,
		M = 0.36	M = 0.53	M = 0.5
Currently smokes	0.46	*n* = 0.81,	*n* = 0.91,	*n* = 0.88,
		Y = 0.17	Y = 0.09	Y = 0.12
Height	0.66	Mean: 167.33	Mean: 168.3	Mean: 170.02
		Median: 168	Median: 173	Median: 170
		Std.Dev: 7.78	Std.Dev: 7.78	Std.Dev: 10.04
		Min: 150	Min: 0	Min: 150
		Max: 185	Max: 186	Max: 188
Caesarean	0.46	*n* = 0.81, Y = 0.17	*n* = 0.91, Y = 0.09	*n* = 0.88, Y = 0.12
Eat yogurt at least 4 days/week	0.07	*n* = 0.74, Y = 0.26	*n* = 0.91, Y = 0.09	*n* = 0.78, Y = 0.22
Eat bread at least 4 days/week	0.15	*n* = 0.33, Y = 0.67	*n* = 0.19, Y = 0.81	*n* = 0.17, Y = 0.83
Eat pasta at least 4 days/week	0.06	*n* = 0.29, Y = 0.71	*n* = 0.15, Y = 0.85	*n* = 0.34, Y = 0.66
Eat dairy products at least 4 days/week	0.04	*n* = 0.36, Y = 0.64	*n* = 0.4, Y = 0.6	*n* = 0.59, Y = 0.41
Eat fruit and vegetables at least 4 days/week	0.01	*n* = 0.14, Y = 0.86	*n* = 0.34, Y = 0.66	*n* = 0.14, Y = 0.86
Eat meat at least 4 days/week	0.33	*n* = 0.24, Y = 0.76	*n* = 0.3, Y = 0.7	*n* = 0.38, Y = 0.62
Eat fish at least 4 days/week	0.45	*n* = 0.74, Y = 0.26	*n* = 0.83, Y = 0.17	*n* = 0.74, Y = 0.26
Eat cereals at least 4 days/week	0.02	*n* = 0.71, Y = 0.29	*n* = 0.89, Y = 0.11	*n* = 0.67, Y = 0.33
Eat legumes at least 4 days/week	0.002	*n* = 0.62, Y = 0.38,	*n* = 0.90, Y = 0.10,	*n* = 0.84, Y = 0.16,
Drink coffee at least 4 days/week	0.20	*n* = 0.14, Y = 0.86	*n* = 0.28, Y = 0.72	*n* = 0.28, Y = 0.72
Physical activity	0.10	*n* = 0.4, Y = 0.6	*n* = 0.56, Y = 0.44	*n* = 0.36, Y = 0.64

## Appendix B

**Table A2:** Demographic and habits-related parameters of the cohort. The *p*-values refer to the Kruskall–Wallis test and Fisher’s test between the Montreal E scale and the numeric or categorical variables, respectively. *n* = no, Y = yes. The variable in bold was significantly unbalanced between HC (42 samples), E1 (9 samples), E2 (18 samples) and E3 (28 samples) groups.

	*p*-Value	HC	E1	E2	E3
Height	0.39	Mean: 167.73	Mean: 173.33	Mean: 169.28	Mean: 169.36
		Median: 168	Median: 175	Median: 168.5	Median: 171
		Std.Dev: 7.92	Std.Dev: 10.69	Std.Dev: 10.25	Std.Dev: 9.96
		Min: 150	Min: 158	Min: 155	Min: 150
		Max: 185	Max: 188	Max: 185	Max: 188
Drink coffee at least 4 days/week	0.45	*n* = 0.16, Y = 0.84	*n* = 0.22, Y = 0.78	*n* = 0.22, Y = 0.78	*n* = 0.32, Y = 0.68
Eat meat at least 4 days/week	0.33	*n* = 0.23, Y = 0.77	*n* = 0.33, Y = 0.67	*n* = 0.44, Y = 0.56	*n* = 0.36, Y = 0.64
Eat cereals at least 4 days/week	0.47	*n* = 0.73, Y = 0.27	*n* = 0.56, Y = 0.44	*n* = 0.78, Y = 0.22	*n* = 0.61, Y = 0.39
Physical activity	0.72	*n* = 0.39, Y = 0.61	*n* = 0.56, Y = 0.44	*n* = 0.33, Y = 0.67	*n* = 0.36, Y = 0.64
Age	0.13	Mean: 38.98	Mean: 39.22	Mean: 44.28	Mean: 43.18
		Median: 34.5	Median: 40	Median: 46.5	Median: 41
		Std.Dev: 16.33	Std.Dev: 10.31	Std.Dev: 13.37	Std.Dev: 15.51
		Min: 22	Min: 25	Min: 20	Min: 19
		Max: 72	Max: 58	Max: 69	Max: 68
Eat fruit and vegetables at least 4 days/week	0.67	*n* = 0.14, Y = 0.86	Y = 1	*n* = 0.17, Y = 0.83	*n* = 0.18, Y = 0.82
Currently smokes	0.49	*n* = 0.68, Y = 0.32	*n* = 0.89, Y = 0.11	*n* = 0.72, Y = 0.28	*n* = 0.82, Y = 0.18
Gender	0.54	F = 0.64,	F = 0.44,	F = 0.5,	F = 0.5,
		M = 0.36	M = 0.56	M = 0.5	M = 0.5
Eat dairy products at least 4 days/week	0.01	*n* = 0.36, Y = 0.64	*n* = 0.56, Y = 0.44	*n* = 0.83, Y = 0.17	*n* = 0.46, Y = 0.54
Eat legumes at least 4 days/week	0.07	*n* = 0.64, Y = 0.36	*n* = 0.78, Y = 0.22	*n* = 0.94, Y = 0.06	*n* = 0.79, Y = 0.21
Eat bread at least 4 days/week	0.23	*n* = 0.34, Y = 0.66	*n* = 0.11, Y = 0.89	*n* = 0.22, Y = 0.78	*n* = 0.14, Y = 0.86
Caesarean section	0.96	*n* = 0.82, Y = 0.16	*n* = 0.89, Y = 0.11	*n* = 0.83, Y = 0.17	*n* = 0.89, Y = 0.11
Eat pasta at least 4 days/week	0.71	*n* = 0.3, Y = 0.7	*n* = 0.44, Y = 0.56	*n* = 0.39, Y = 0.61	*n* = 0.29, Y = 0.71
Eat fish at least 4 days/week	0.71	*n* = 0.75, Y = 0.25	*n* = 0.89, Y = 0.11	*n* = 0.72, Y = 0.28	*n* = 0.68, Y = 0.32
Eat yogurt at least 4 days/week	0.95	*n* = 0.75, Y = 0.25	*n* = 0.78, Y = 0.22	*n* = 0.83, Y = 0.17	*n* = 0.75, Y = 0.25

## Appendix C

**Table A3:** Demographic and habits-related parameters of the cohort. The *p*-values refer to the Kruskall–Wallis test and Fisher’s test between the Montreal B scale and the numeric or categorical variables, respectively. *n* = No, Y = yes. The variables in bold were unbalanced between the HC (42 samples), B1 (27 samples), B2 (22 samples) and B3 (3 samples) groups.

	*p*-Value	HC	B1	B2	B3
Height	0.28	Mean: 167.33	Mean: 170.07	Mean: 166.27	Mean: 167.5
		Median: 168	Median: 173	Median: 176	Median: 165
		Std.Dev: 7.78	Std.Dev: 9.77	Std.Dev: 38.34	Std.Dev: 13
		Min: 150	Min: 153	Min: 0	Min: 155
		Max: 185	Max: 185	Max: 186	Max: 185
Drink coffee at least 4 days/week	0.02	*n* = 0.14, Y = 0.86	*n* = 0.33, Y = 0.67	*n* = 0.14, Y = 0.86	*n* = 0.75, Y = 0.25
Eat meat at least 4 days/week	0.33	*n* = 0.24, Y = 0.76	*n* = 0.26, Y = 0.74	*n* = 0.41, Y = 0.59	Y = 1
Eat cereals at least 4 days/week	0.09	*n* = 0.71, Y = 0.29	*n* = 0.85, Y = 0.15	*n* = 0.95, Y = 0.05	*n* = 0.75, Y = 0.25
Physical activity	0.03	*n* = 0.4, Y = 0.6	*n* = 0.67, Y = 0.33	*n* = 0.55, Y = 0.45	Y = 1
Age	0.03	Mean: 38.02	Mean: 42.41	Mean: 42.82	Mean: 40.25
		Median: 32.5	Median: 45	Median: 41.5	Median: 41
		Std.Dev: 15.95	Std.Dev: 13.84	Std.Dev: 11.39	Std.Dev: 16.82
		Min: 22	Min: 19	Min: 26	Min: 21
		Max: 72	Max: 67	Max: 64	Max: 58
Eat fruit and vegetables at least 4 days/week	0.09	*n* = 0.14, Y = 0.86	*n* = 0.3, Y = 0.7	*n* = 0.41, Y = 0.59	*n* = 0.25, Y = 0.75
Currently smokes	0.08	*n* = 0.67, Y = 0.33	*n* = 0.48, Y = 0.52	*n* = 0.73, Y = 0.23	*n* = 1
Gender	0.07	F = 0.64,	F = 0.56,	F = 0.32,	F = 0.75,
		M = 0.36	M = 0.44	M = 0.68	M = 0.25
Eat dairy products at least 4 days/week	0.45	*n* = 0.36, Y = 0.64	*n* = 0.41, Y = 0.59	*n* = 0.32, Y = 0.68	*n* = 0.75, Y = 0.25
Eat legumes at least 4 days/week	0.00	*n* = 0.62, Y = 0.38	*n* = 0.85, Y = 0.15	*n* = 0.95, Y = 0.05	*n* = 0.75, Y = 0.25
Eat bread at least 4 days/week	0.02	*n* = 0.33, Y = 0.67	*n* = 0.19, Y = 0.81	*n* = 0.09, Y = 0.91	*n* = 0.75, Y = 0.25
Caesarean section	0.4	*n* = 0.81, Y = 0.17	*n* = 0.96, Y = 0.04	*n* = 0.82, Y = 0.18	*n* = 1
Eat pasta at least 4 days/week	0.07	*n* = 0.29, Y = 0.71	*n* = 0.07, Y = 0.93	*n* = 0.18, Y = 0.82	*n* = 0.5, Y = 0.5
Eat fish at least 4 days/week	0.65	*n* = 0.74, Y = 0.26	*n* = 0.85, Y = 0.15	*n* = 0.82, Y = 0.18	*n* = 0.75, Y = 0.25
Eat yogurt at least 4 days/week	0.1	*n* = 0.74, Y = 0.26	*n* = 0.89, Y = 0.11	*n* = 0.95, Y = 0.05	*n* = 0.75, Y = 0.25
